# Initiated Chemical Vapor Deposition (iCVD) of Bio-Based Poly(tulipalin A) Coatings: Structure and Material Properties

**DOI:** 10.3390/polym14193993

**Published:** 2022-09-23

**Authors:** Valeria Graur, Adrivit Mukherjee, Khaled O. Sebakhy, Ranjita K. Bose

**Affiliations:** Engineering and Technology Institute Groningen (ENTEG), University of Groningen, Nijenborgh 4, 9747 AG Groningen, The Netherlands

**Keywords:** Sustainability, iCVD, polymer coatings, bio-based, thermal stability, Butyrolactones

## Abstract

A solvent-free route of initiated chemical vapor deposition (iCVD) was used to synthesize a bio-renewable poly(α-Methylene-γ-butyrolactone) (PMBL) polymer. α-MBL, also known as tulipalin A, is a bio-based monomer that can be a sustainable alternative to produce polymer coatings with interesting material properties. The produced polymers were deposited as thin films on three different types of substrates—polycarbonate (PC) sheets, microscopic glass, and silicon wafers—and characterized via an array of characterization techniques, including Fourier-transform infrared (FTIR), proton nuclear magnetic resonance spectroscopy (^1^H NMR), ultraviolet visible spectroscopy (UV–vis), differential scanning calorimetry (DSC), size-exclusion chromatography (SEC), and thermogravimetric analysis (TGA). Optically transparent thin films and coatings of PMBL were found to have high thermal stability up to 310 °C. The resulting PMBL films also displayed good optical characteristics, and a high glass transition temperature (*T_g_*~164 °C), higher than the *T_g_* of its structurally resembling fossil-based linear analogue-poly(methyl methacrylate). The effect of monomer partial pressure to monomer saturation vapor pressure (*P_m_/P_sat_*) on the deposition rate was investigated in this study. Both the deposition rate and molar masses increased linearly with Pm/Psat following the normal iCVD mechanism and kinetics that have been reported in literature.

## 1. Introduction

Synthetic polymers are a class of very important and versatile materials [1]. Current environmental constraints along with the depletion of fossil fuels necessitate finding alternative sustainable solutions, and the use of greener materials [2,3]. Additionally, an emerging ultimate interest is to replace conventional monomers (derived from fossil fuels) with bio-renewable substitutes (derived from biomass) to produce sustainable polymers and natural alternatives to petroleum-sourced plastics [4]. The production of polymers using bio-based monomers via environmentally friendly and safe processes is the way to truly “green” polymeric material [2,3,4].

**Figure 1:**
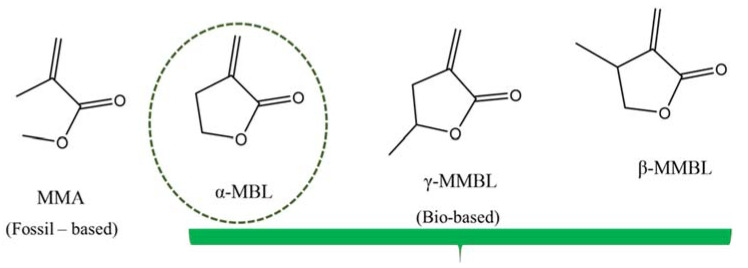
Monomer structures: fossil-based (MMA) and bio-based γ-butyrolactone monomers (α-MBL, γ-MMBL, and β-MMBL).

Biomass-derived monomers based on a five-membered γ-butyrolactone ring and possessing a vinyl moiety allowing radical polymerization (Figure 1) represent suitable and attractive candidates to replace sources of fossil origin [5]. Those bio-based monomers are cyclic analogues of methyl methacrylate (MMA) bearing both a vinyl group and lactone ring, as depicted in Figure 1.

From a synthetic standpoint, those monomers can be cost-effectively produced from itaconic acid (IA), which is an intermediate of biomass processing such as fermentation of corn or rice [5,6,7,8,9]. Thus, this is a sustainable process to produce those bio-renewable monomers. Furthermore, in 2004, DuPont commercialized a two-step catalytic process for the production of MMBL monomers from levulinic acid, which is a biomass intermediate [10].

Butyrolactone sustainable polymers have been previously synthesized via a wide range of free-radical polymerization (FRP) techniques, such as conventional FRP [11,12,13,14,15,16], atom-transfer radical polymerization (ATRP) [17], and reversible-addition fragmentation chain transfer (RAFT) [5,18,19,20]. Despite the interesting and unique properties of those monomers and their polymers, their polymerization studies are still relatively scarce in the literature and were mainly carried out in highly toxic organic solvents to produce bulk polymers, at low monomer conversions [5].

Among the most famous γ-butyrolactone monomers is α-methylene-γ-butyrolactone (α-MBL, Tulipalin A) [5,11,17,19,21,22], which can be directly derived from tulips (Tulipa gesneriana L) that are widespread in the Netherlands [23]. It has the strongest resemblance to the widely used methyl methacrylate (MMA) monomer and was therefore chosen as the focus of this work. α-MBL has a five-membered lactone ring (Figure 1), which can be used for hydrolyzable drug bonding as well as in co-polymerization by ring-opening polymerizations (ROP) [24]. The lactone ring can thus be used in analogous reactions to make new bio-based materials with various characteristics [15,19]. Additionally, α-MBL has sites where specific biomolecules can be attached for targeted drug delivery, which would serve as an interesting research pathway for novel drug delivery systems [25]. Compared to its linear analogue MMA, α-MBL is nearly planar, more polar than MMA, and is more reactive since its double bond is more accessible given the less steric interactions with the neighboring groups [11,26]. The most commonly reported free-radical polymerization of α-MBL took place via the reaction of the vinyl double bond in the presence of a free-radical initiator [11]. α-MBL polymerized to poly(α-methylene-γ-butyrolactone) (PMBL), which showed advanced and unique properties such as high optical transparency, high mechanical strength, scratch resistance, and improved physical resistance [5,11,15,27]. Moreover, PMBL was found to be very resistant towards solvent attack and thus insoluble in all organic solvents except dimethyl sulfoxide (DMSO) and dimethyl formamide (DMF) [26]. This useful property provides various applications to the polymer since it can be used to aid extra solvent protection and thus offer protection to polymers and substrates that show poor solvent resistance [11,26].

Nowadays, with the continuous growth and development in material science, there has always been a dire need for the production of advanced thin-film materials [28], and polymer coatings [29] with well controlled thicknesses. Thin films are known to find potential applications in surface coatings, photonics, surface modification, optical devices, electronics, environmental applications, and other high technology applications [29,30]. Among the most attractive processes to make polymer films and coatings is initiated chemical vapor deposition (iCVD) [31,32,33,34,35,36,37,38,39]. Apart from being solvent-free and an environmentally friendly procedure, iCVD provides the compelling advantage of combining polymerization and coating in a single step [31,32,33,34,35,36]. It has multiple benefits, especially when compared to other CVD processes, such as iCVD being lower in energy consumption than hot filament chemical vapor deposition (HFCVD), for example [40,41]. 

Hence, by coupling the advantages of iCVD with bio-based monomers in this work, we envisage for the first time the synthesis of sustainable Poly(tulipalin A) coatings having advanced material properties, such as high glass transition temperature (*T_g_*), high thermal stability, optical transparency, and solvent resistance.

## 2. Experimental Part

### 2.1. Materials

**Scheme 1:**
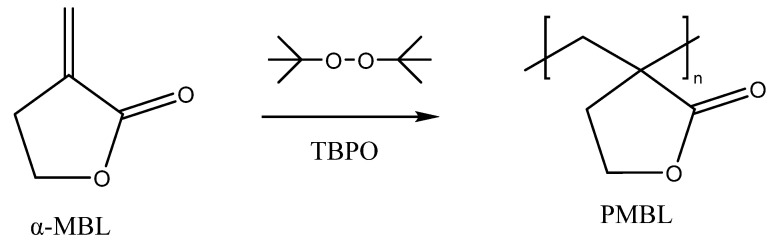
Free radical polymerization of α-MBL via iCVD.

Alpha-Methylene-Gamma-Butyrolactone monomer (α-MBL/Tulipalin-A > 95% purity, CAS: 547-65-9) was purchased from TCI Chemicals and used as received without further purification. Di-tert-butyl peroxide (TBPO, 98% purity, CAS: 110-05-4, Sigma Aldrich, St. Louis, MO, USA) was used as a free-radical initiator. Deuterated dimethyl sulfoxide (DMSO-d_6_) (CAS: 2206-27-1, Sigma-Aldrich) was used as the solvent for ^1^H-NMR measurements. Dimethyl formamide (DMF) with 0.01mM LiBr was used as the GPC solvent for molecular weight determination. Microscopic glass slides (Knittel Glass, 76 × 26 mm dimension), FTIR transparent Silicon wafers (University Wafers Inc., Boston, MA, USA, one side polished, 525 +/− 25 µm thickness), and polycarbonate sheets (123-3D, Almere, Netherlands., 1.27 mm thickness) were used as received. The iCVD polymerization is illustrated in Scheme 1.

### 2.2. Process Conditions for iCVD of PMBL

Poly(α-methylene-γ-butyrolactone) (PMBL) was synthesized and deposited as thin films on silicon wafers, glass slides, and polycarbonate sheets via iCVD using TBPO as the initiator. A detailed description and schematic of the custom-built iCVD reactor setup are illustrated in Figures S1 and S2, and Section S1, of the supporting information. Briefly, during a typical iCVD run, the reactant delivery lines were heated to 110 °C, and the main reaction chamber was maintained at 40 °C. The monomer was heated to 75 °C in a glass jar to ensure enough vapor pressure for maintaining constant flows during the reaction, while the initiator jar was kept at room temperature. The monomer and the initiator flows were held at a steady rate of 1.2 sccm and 0.6 sccm, respectively, using precision needle valves. Therefore, for all depositions, the ratio of monomer to initiator flows (M)/(I) was held at a constant value of 0.5. The nichrome filament array used for the thermal decomposition of the initiator was held at 250–270 °C for all the iCVD runs. The depositions were performed on various substrates attached to a custom-built stage. The stage temperature was controlled using a recirculating chiller (Minichiller 600-H Olé, Huber, Offenburg, Germany) and monitored using standard thermocouples. The pressure inside the main reaction chamber was monitored using a manometer (Baratron, MKS Instruments, Andover, MA, USA).

The deposition kinetics of iCVD of PMBL was explored by collecting two sets of data to investigate the effect of varying *P_m_*/*P_sat_* on the deposition rate. *P_m_*/*P_sat_* is analogous to the monomer concentration in liquid-phase polymerization. The detailed parameters of deposition are summarized in Table 1. In the first series of depositions (the pressure series), the total pressure in the main reaction chamber varied from 0.2–0.4 Torr while keeping all other parameters constant, to find out the influence of changing Pm on the deposition rate (Samples P1–P4). To extract the activation energy (*E*_a_) for iCVD of PMBL, the second set of depositions (the substrate temperature series) was carried out by varying the substrate temperature from 25–40 °C (Samples T1–T4). The reactor pressure was also varied to arrive at the final *P_m_/P_sat_* values for this set of depositions. The *P_sat_* values for MBL at different temperatures were calculated using the Clausius–Clapeyron equation:

(1) $$\ln\left(P_{sat}\right)=\frac{A}{T_{s}}+B$$

where *T_s_* is the substrate temperature; A = −9732.60 and B = 28.97 were calculated from the slope and intercept of the plot of ln *P_sat_* versus 1/*T_s_*, respectively; and $P_{sat}\;$is the saturation vapor pressure of the monomer (MBL) at the particular substrate temperature (*T_s_*).

The *P_m_* value was calculated from the following equation:

(2) $$P_{m}=\frac{x_{m}}{x_{m}+x_{i}}\times P_{reactor}$$

where *P_m_* represents the monomer partial pressure, and *x_m_* = 1.2 sccm and *x_i_* =0.6 sccm display the monomer and initiator flow rates, respectively. The pressure inside the reactor was chosen as the variation parameter.

Furthermore, a third set of depositions were carried out for comparatively longer durations to obtain thicker samples of the synthesized iCVD PMBL for the characterization experiments (samples C1–C3). The experimental conditions are outlined in Table 1 (the characterization series).

The thickness of all polymer coatings was measured using a profilometer (Bruker, DektakXT, Billerica, MA, USA) employing Vision64 software. For all iCVD reactions, monomer conversion was confirmed by FTIR, ^1^H NMR, and SEC measurements. The thermal properties of the produced coatings were assessed by TGA and DSC to determine decomposition temperature and *T_g_*, respectively. The transparency of all coatings was checked using UV–vis. The molecular-weight distributions were determined by SEC, and the mechanical properties were analyzed via the nanoindentation technique. All details on the characterization techniques are included in Section S3 of the supporting information.

## 3. Results and Discussion

The iCVD experiments were carried out at different *P_m_*/*P_sat_* (0.35, 0.44, 0.52, 0.61, and 0.70), as previously explained in the experimental section. Details on experimental conditions are included below in Table 1.

**Figure 2:**
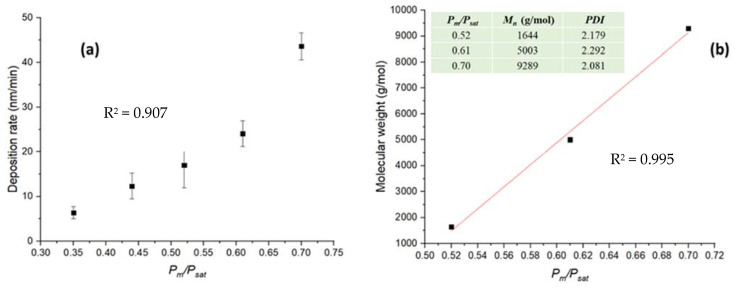
(**a**) PMBL deposition rate as a function of *P_m_*/*P_sat_* and (**b**) *P_m_*/*P_sat_* versus number-average molar mass (*M_n_*).

Samples (P1, P2, P3, P4, and P5) were prepared by iCVD for a duration of 40 min. Those samples were used to determine a relationship between the rate of deposition and the reactor pressure (P). As can be observed from the data in Table 1 (pressure series), an increase in P (total pressure) results in an increase in the surface monomer concentration and consecutively an increase in *P_m_*/*P_sat_* and the deposition rate, which is also clearly depicted in Figure 2a.

As shown in Figure 2a, an increase in *P_m_*/*P_sat_*, which is considered analogous to monomer concentration in bulk-phase polymerization, leads to an increase in the rate of PMBL deposition, as predicted. The fit was observed to follow linear behavior and a correlation coefficient (R^2^) of 0.9074. The kinetics of PMBL deposition are in agreement with the kinetic model previously reported by Lau and Gleason [35]. 

Furthermore, Figure 2b shows that increasing *P_m_*/*P_sat_* results in a linear increase in the polymer molar mass. Hence, increasing *P_m_*/*P_sat_* has the benefit of a simultaneous increase in both deposition rate and molar mass. The range of the reported polydispersity index (PDI) values is between 2.1 and 2.3, which corresponds to the results of PMBL obtained via conventional free radical polymerization stating a PDI of 2.0. Detailed molar mass distributions are provided in the supplementary material section (Figure S6).

**Figure 3:**
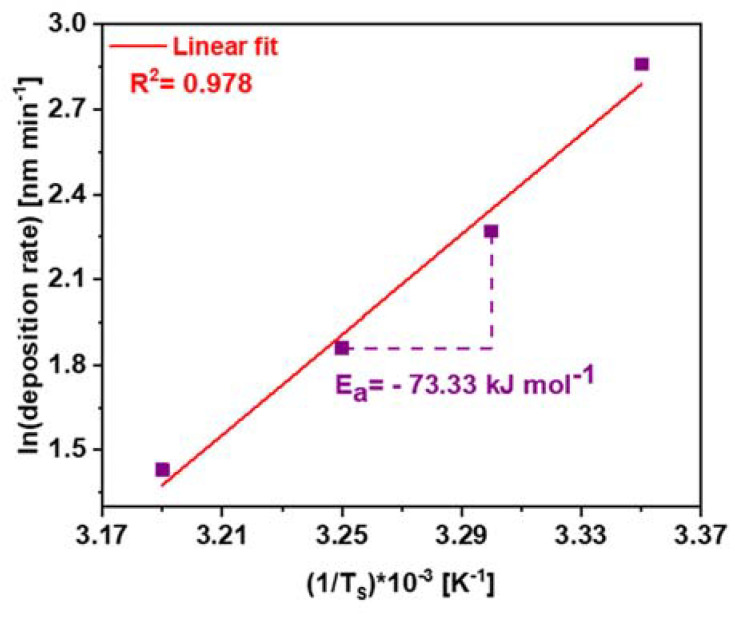
Arrhenius plot of deposition rate as a function of varying substrate temperature (*T*_s_) to derive the activation energy (*E*_a_) for iCVD of PMBL.

In the second series of iCVD of PMBL (substrate temperature series—see Table 1), the deposition rate was measured with varying substrate temperatures (*T*_S_). The experimental activation energy was determined by regressing the data (Figure 3) and using Equation (2) [35]

(3) $$E_{a}=-R\left[\frac{\partial \left(\ln DR\right)}{\partial \left(\frac{1}{T_{S}}\right)}\right]$$

where *E*_a_ is the experimental activation energy, R is the universal gas constant, and *DR* is the deposition rate at a particular substrate temperature (*T*_s_).

Consistent with previous reports in the literature, the deposition kinetics of iCVD PMBL were found to be adsorption-limited, as strongly evidenced by faster polymerization rates of PMBL with decreasing substrate temperatures and indicating an apparent negative value for overall activation energy [34,35]. This can be attributed to a higher surface concentration of monomers at lower substrate temperatures. According to the Arrhenius relationship (Equation (2)), the slope of the curve in Figure 3 represents −*E*_a_/R; hence, the overall activation energy for the iCVD of PMBL was determined to be 73.3 kJ mol^−1^. The experimentally determined value of the overall activation energy for iCVD of PMBL was found to bear a remarkably close match to the reported value of 76.1 kJ mol^−1^ in the literature for free radical polymerization in the solution phase [12]. The good agreement strongly supports the existing theory of surface-driven iCVD rate kinetics being adsorption-limited and closely analogous to those of free radical polymerization in the solution phase [34,35].

**Figure 4:**
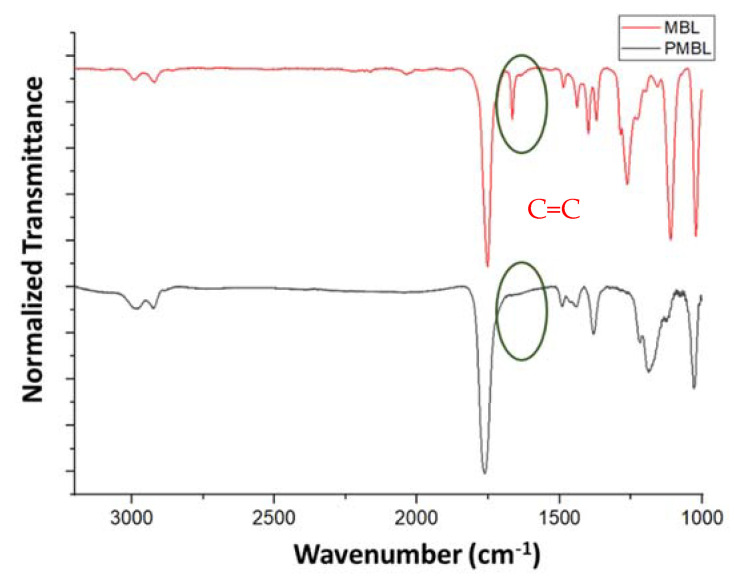
A comparison between FTIR spectra of α-MBL monomer and PMBL (PMBL sample (P3 in Table 1) produced by iCVD at *P_m_*/*P_sat_* = 0.52).

The FTIR measurements of both the monomer and the polymer were recorded to identify structural differences and confirm the successful conversion of α-MBL to PMBL, as depicted in Figure 4. A peak between 2840 and 3000 cm^−1^ was assigned to C-H stretching in both the monomer and the polymer spectra. The broadest peak at 1760 cm^−1^ for both α-MBL and PMBL spectra was attributed to the C=O lactone stretching, and it corresponds to the reported C=O stretching in gamma-butyrolactone moieties [19]. This peak was compared to previously reported IR spectra of PMBL obtained via solution-phase RAFT polymerization, and it was confirmed that polymerization occurred by vinyl addition rather than ring opening [3,11]. The 1664 cm^−1^ peak attributed to the C=C stretching in the FTIR spectrum of the monomer (α-MBL) is no longer observed in the polymer spectrum, which proves the completion of the polymerization reaction without the presence of any remaining monomer [3,11,19]. Another peak between 1440 and 1500 cm^−1^ was assigned to methylene C-H bending [11]. The bands corresponding to the ester C-O bond were observed between 1100 and 1200 cm^−1^ for both the monomer and the polymer [19]. Detailed FTIR spectra for different polymer samples are incorporated in the supplementary material section (Figure S4).

**Figure 5:**
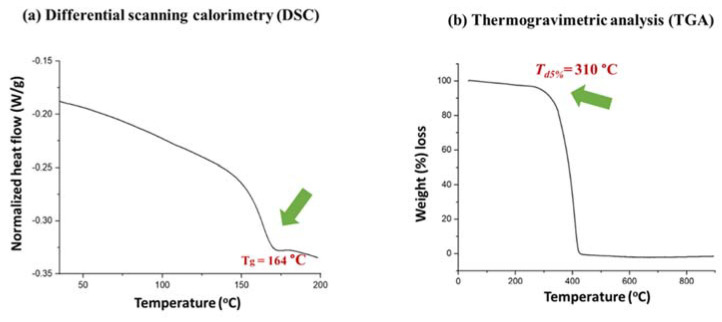
Characterization of thermal properties of PMBL (sample (C4 in Table 1) produced by iCVD at *P_m_*/*P_sat_* = 0.71; *M_n_* = 9289 g/mol and PDI = 2.08): (**a**) DSC: *T_g_* obtained from the exothermic peak of the second heating cycle and (**b**) TGA.

DSC characterization was performed to determine the glass transition temperature (*T_g_*) of PMBL polymers, as shown in Figure 5a. The molecular weight and the obtained *T_g_* of different PMBL samples are also presented in Table 2. It can be observed that an increase in molecular weight caused a significant increase in the glass transition temperature, and the highest *T_g_* value was attained for the highest *M_n_* polymer, which was produced at the highest deposition rate (*P_m_*/*P_sat_* = 0.70). This dependence of the glass transition temperature on the molecular weight is explained by the Flory–Fox equation and corresponds to the expected trend [42]. The *T_g_* of PMBL obtained in this work is lower than the values of 190–195°C reported in the literature for PMBL prepared by solution polymerization [3,5]. The 190 °C *T_g_* was determined for a sample that displayed a molecular weight of g/mol, which is much higher than the molecular weights of the samples considered for the thermal characterizations in this work and thus explains this difference in *T_g_*. Furthermore, this high glass transition temperature being higher than that of PMMA can allow the use of PMBL in applications where PMMA or other alternatives [13,14] are not suitable, as previously mentioned in literature [5,19].

The thermogram of PMBL showed one-step decomposition behavior between 250 and 400 °C, as depicted in Figure 5b. The high thermal stability of PMBL produced by iCVD was confirmed by TGA through the decomposition of only 5 wt.% of the polymer at 310 °C (i.e., T_d5%_), while the complete thermal decomposition took place at 400 °C. This behavior is in good agreement with previously reported thermal stability data for PMBL [5,11,19]. This high thermal stability of PMBL is attributed to the lactone ring, which provides the polymer chain with extra rigidity and thus enhances its thermal stability [3,4,5,11,26].

**Figure 6:**
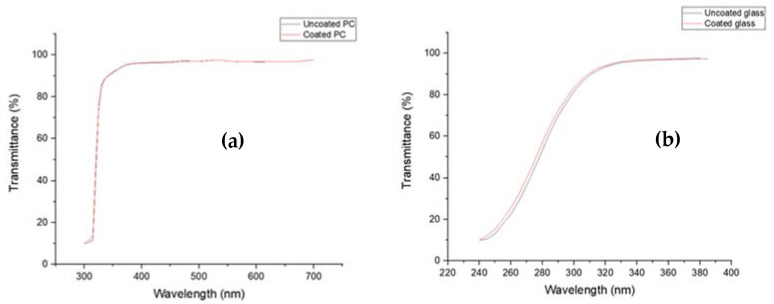
Transparency analysis tests via UV–vis of PMBL films on (**a**) PC substrates (coating thickness = 2.6 µm) and (**b**) glass substrate (coating thickness = 4.2 µm).

In order to analyze the possible range of applications for these new PMBL coatings, the transparency was measured using UV–vis spectroscopy. It was done by recording and plotting the wavelength versus light transmittance. This method was previously used for thin films on substrates to prove their transparency [43,44]. The characterization was performed using polycarbonate (Figure 6a) since it is an amorphous thermoplastic, as well as by using microscopic glass substrates (Figure 6b).

The UV–vis tests showed no significant difference in terms of absorbance for the coated and uncoated substrates (PC and glass), which means that the PMBL coatings are transparent and therefore suitable for coating objects requiring unobstructed visibility. The high optical transparency of PMBL thin films is a very beneficial characteristic, especially for the range of their potential applications.

Figures S1 and S2 (the iCVD schematic and setup), Figure S3 (the effect of substrate position on deposition rate), Figure S4 (the compiled FTIR spectra of PMBL samples), Figure S5 (the ^1^H NMR spectra of PMBL), Figure S6 (the MWDs of PMBL samples by SEC), Figures S7–S10 (the nanoindentation tests), and Figure S11 (the DSC data for samples P3 and P4) are shown.

## 4. Conclusions and Future Perspectives

In summary, novel, sustainable, thermally stable, and transparent coatings of PMBL were prepared in a facile manner via iCVD. The kinetics of deposition were found to have a linear relationship with *P_m_*/*P_sat_*, as previously reported in the literature studies. Additionally, increasing the reaction chamber pressure increased both the deposition rates and the molar masses simultaneously. The coatings thickness can be easily controlled using iCVD. This work opens the door for exploring sustainable bio-based coatings that can withstand high temperatures and are solvent and scratch-resistant. Furthermore, other types of butyrolactone monomers (e.g., γ-MMBL and β-MMBL) can be investigated by iCVD.

## Figures and Tables

**Table 1:** Experimental conditions for iCVD of MBL on Si wafer substrate. The pressure series corresponds to Figure 2, and the substrate temperature series corresponds to Figure 3.

**Pressure series**							
**Sample Name**	**Monomer Flow (*F_M_*)** **(sccm)**	**Initiator Flow (*F_I_*)** **(sccm)**	**Substrate Temperature** **(** **°** **C)**	**Total Pressure (*P*)** **(mTorr)**	**Deposition Time** **(min)**	***P_m_/P_sat_***	**Mean Deposition Rate** **(nm min^−1^)**
P1	1.2	0.6	40	0.20	40	0.35	6.4
P2	1.2	0.6	40	0.25	40	0.45	12.3
P3	1.2	0.6	40	0.30	40	0.52	17.0
P4	1.2	0.6	40	0.35	40	0.61	24.1
P5	1.2	0.6	40	0.40	40	0.71	43.6
**Substrate temperature series**							
**Sample Name**	**Monomer Flow (*F_M_*)** **(sccm)**	**Initiator Flow (*F_I_*)** **(sccm)**	**Substrate Temperature** **(** **°** **C)**	**Total Pressure (P)** **(Torr)**	**Deposition Time** **(min)**	***P_m_*/*P_sat_***	**Mean Deposition Rate** **(nm min^−1^)**
T1	1.2	0.6	25	0.08	40	0.63	17.4
T2	1.2	0.6	30	0.11	40	0.51	9.7
T3	1.2	0.6	35	0.15	40	0.42	6.4
T4	1.2	0.6	40	0.20	40	0.34	4.2
**Characterization series**							
**Sample Name**	**Monomer Flow (*F_M_*)** **(sccm)**	**Initiator Flow (*F_I_*)** **(sccm)**	**Substrate Temperature** **(** **°** **C)**	**Total Pressure (P)** **(Torr)**	**Deposition Time** **(min)**	***P_m_*/*P_sat_***	
C1	1.2	0.6	40	0.30	190	0.52	
C3	1.2	0.6	40	0.35	300	0.61	
C4	1.2	0.6	40	0.40	240	0.71	

**Table 2:** The effect of PMBL molar mass (*M_n_*) on glass transition temperature (*T_g_*).

Sample Name	*P_m_*/*P_sat_*	*M_n_* (g/mol)	*T_g_* (°C)
P3	0.52	1644	105
P4	0.61	5003	153
P5	0.70	9289	164

## Data Availability

The data presented in this study are available in  Supplementary Materials.

## Supplementary Materials

The following supporting information can be downloaded at: https://www.mdpi.com/article/10.3390/polym14193993/s1, Figure S1: Schematic representation of iCVD setup; Figure S2: iCVD laboratory setup at University of Groningen, Netherlands; Figure S3: Preliminary experiment: Effect of the substrate position on the iCVD stage on the rate of deposition; Figure S4: Compiled FTIR spectra of all the polymer spectra (*P_m_*/*P_sat_* = 0.3 – 0.7); Figure S5: Stack plot for the ^1^H NMR spectrum of PMBL samples; Figure S6: MWDs of PMBL (obtained via SEC using PMMA standards): For the samples P0.52a, P0.61a, P0.70a in Table 1); Figure S7: Mechanical properties: nanoindentation data – Displacement vs load curve; Figure S8: Mechanical properties: Displacement vs load diagram (five randomly chosen curve compilation); Figure S9: Nanoindentation results for multiple samples: PMBL modulus; Figure S10: Nanoindentation results for multiple samples: PMBL hardness; Figure S11: DSC data for samples P3 and P4; Table S1: Peak assignments for FTIR spectrum; Table S2: Nanoindentation results.
