# Supplementary material for: Initiated Chemical Vapor Deposition (iCVD) of Bio-Based Poly(tulipalin A) Coatings: Structure and Material Properties
Source: Polymers (Basel). 2022 Sep 23;14(19):3993. doi: 10.3390/polym14193993 (PMC9572868; doi:10.3390/polym14193993)
Supplement: Supplementary file 1 [file polymers-14-03993-s001.zip › polymers-1911641-supplementary.pdf]

Initiated Chemical Vapor Deposition (iCVD) of Bio-based Poly(tulipalin A) Coatings: Structure and Material Properties

Valeria Graur[1], Adrivit Mukherjee[2], Khaled O. Sebakhy[3*], Ranjita K. Bose[4*]

Engineering and Technology Institute Groningen (ENTEG), Polymer Product Technology, University of Groningen, Nijenborgh 4, Groningen 9747 AG, The Netherlands

Correspondance Emails: k.o.sebakhy@rug.nl, r.k.bose@rug.nl

S.1. Schematic and description of iCVD setup

All experiments were conducted in an initiated chemical vapor deposition reaction setup divided into three different sections: gas delivery system, main reaction chamber and gas exhaust system. A schematic representation, as well as a picture of the real experimental setup, are depicted in Figure S1 and Figure S2, respectively. Gas delivery section consists of gas lines equipped with screw and needle valves that allow the flow control of the reactants (α-MBL and TBPO) as well as temperature controllers and heaters. The main reaction chamber contains a stage attached to an outside cooling source and a Nichrome filament connected to an outside power supply. The downstream of the reactor includes a gas exhaust system that consists of a nitrogen trap and a copper mesh to protect the pump by adsorbing any exiting waste vapours.

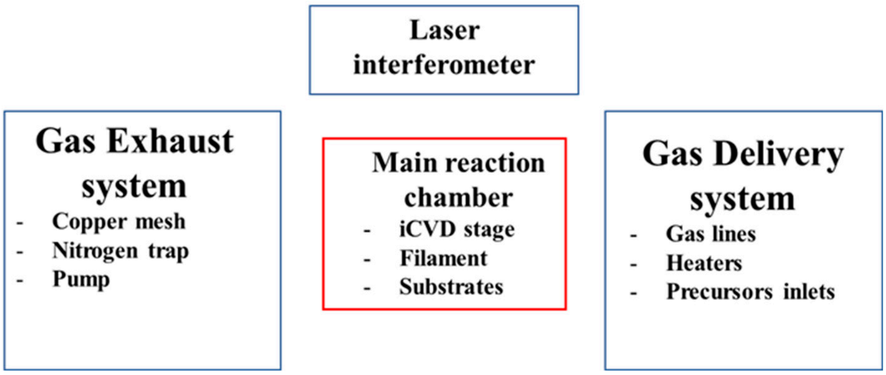

Figure S1. Schematic representation of iCVD setup.

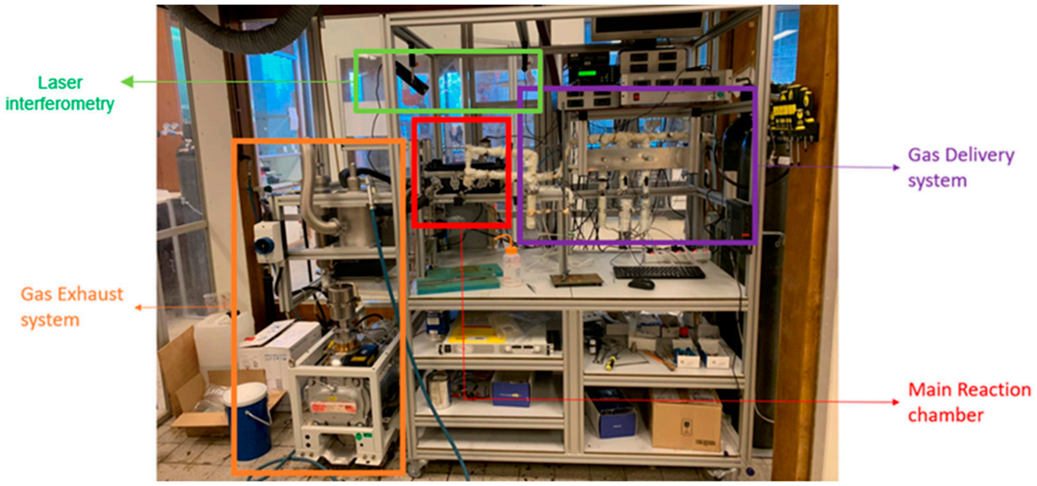

Figure S2. iCVD laboratory setup at University of Groningen, Netherlands.

iCVD experiments were done at a stage temperature of 40 °C and a filament temperature of 280 °C for TBPO initiator thermal decomposition. The studied P_m/P_{sat} parameters were obtained by varying reactor pressure (P), mainly because of convenience, and due to the presence of the butterfly valve in the setup, which allows for precise and accurate control of this parameter. The pressures (P) at which experiments were performed were 0.20, 0.25, 0.30, 0.35, 0.40 Torr in order to study the P_m/P_{sat} parameters of 0.35, 0.44, 0.52, 0.61 and 0.70, respectively. Stage and filament temperatures were monitored by K-type thermocouples attached directly to the corresponding item. Gas line temperature was maintained at 110 °C during the reactions. A heater was attached to the α-MBL monomer jar and set to 70-75 °C to ensure sufficient flow whilst TBPO was maintained at room temperature due to its high volatility. Reactant and initiator flow rates were calibrated as 0.6 and 1.2 sccm (standard cm^3 per minute), respectively, by using needle and screw valves.

S.2. Experimental plan

A preliminary experiment was performed to determine the effect of the substrate position in the stage on the rate of deposition (Figure S3). Multiple substrates were placed on the stage at preliminarily measured distances and the rate of polymer deposition for each was measured. A second experiment confirmed similar results. For all the subsequent experiments, the substrates to be used for deposition rate measurements were placed at the same position, and more exactly the one that previously displayed the highest rate of deposition. The experiments with the condition optimization goal were done for 40 minutes each deposition. The results obtained from these

experiments were used in order to determine the rate of deposition and to plot the relationship between pressure inside the reactor rate of deposition. The experiments with the goal of gathering polymer material for thermal analyses as well as SEC analysis were performed between 190 to 300 minutes to collect enough bulk polymer for characterization. Throughout the reaction, an in-situ interferometer (Type PM100USB serial nr: P2010305, sensor serial nr: 18043035) was kept aligned with a reflective silicon wafer (positioned above the main reaction chamber). This technique was used as a complement to profilometry to assess changes in the coating thickness throughout the course of each reaction by measuring the number of peaks and observing the progress of the deposition.

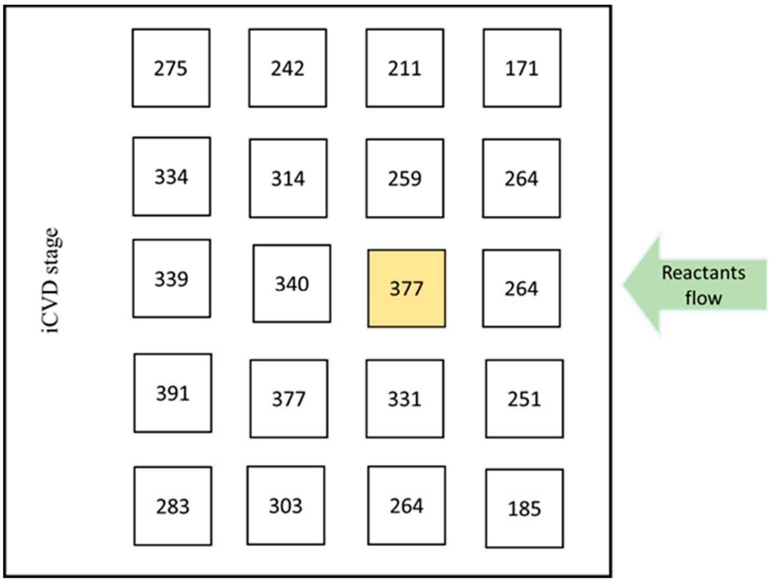

Figure S3. Preliminary experiment: Effect of the substrate position on the iCVD stage on the rate of deposition.

S.2.1. Coating deposition rate and thickness measurement

A profilometer (Bruker, DektakXT) was used to measure the thickness of all the coatings using the *Vision64* software. This was done by making scratches on the substrate using a glass pipette and taking the average of the measurements at three different positions per substrate. The conditions used for all the measurements were: 65.5 µm range; 2000 µm length; 20 seconds duration; and 2 mg stylus force. Division of the obtained thickness value (nm) by the time of

reaction (min) led to the calculation of the rate of deposition. For the deposition rate calculations at each P_m/P_{sat} value, the position of the substrate on the stage was kept constant.

S.3. Characterization techniques

S.3.1. Fourier transform infrared spectroscopy (FTIR)

FTIR was employed to confirm the successful polymerization of the monomer, by inspecting the vinyl bond presence and assessing the polymer composition.[1] For the thin coatings, FTIR was conducted in transmittance mode on a Shimadzu IRTracer-100 by attaching the substrates to a specific sample holder and placing it in the instrument chamber. The measurements were recorded between 600 to 3500 cm^{-1} using 64 scans at a 4 cm^{-1} resolution. All functional groups were assigned as shown in Table S1. A comparative FTIR spectrum is shown in Figure S4.

Table S1. Peak assignments for FTIR spectrum

Wavenumber (cm^{-1})	Peak assignment
3000-2840	C-H
1760	C=O lactone
1440-1500	C-H methylene
1100-1200	C-O ester
1664	C=C (monomer)

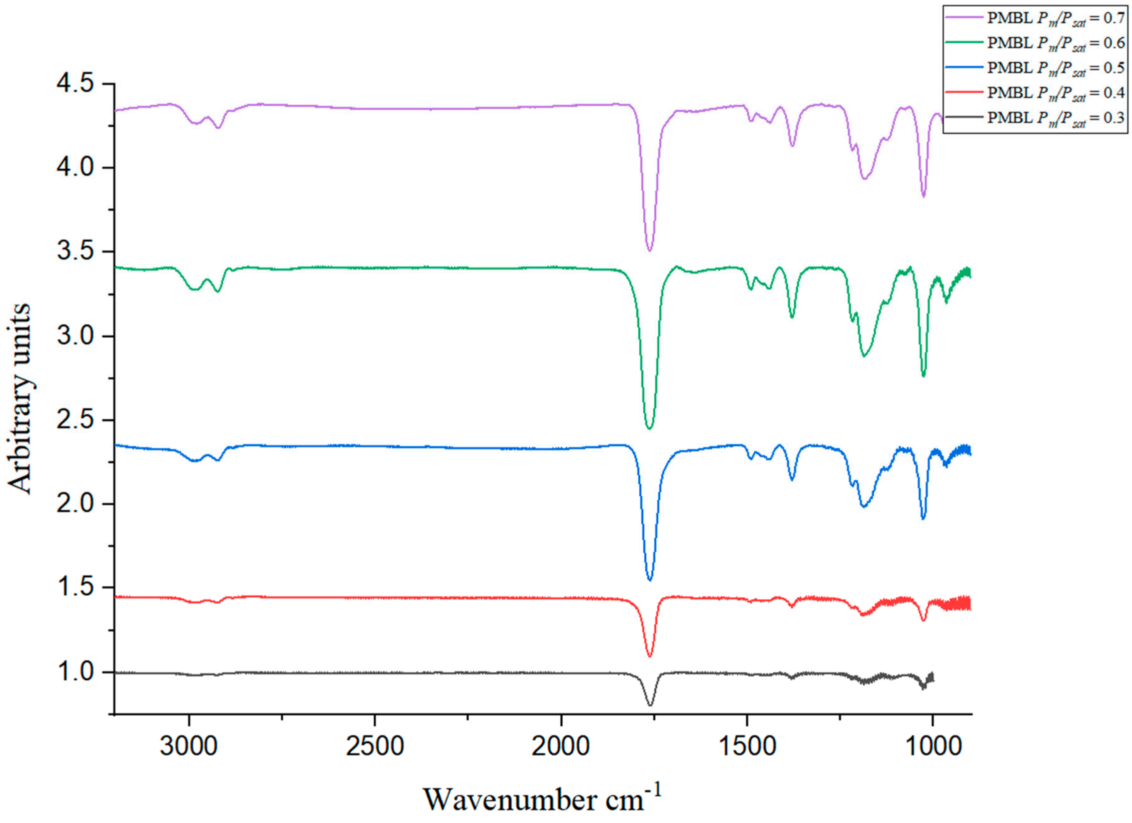

Figure S4. Compiled FTIR spectra of all the polymer spectra (P_m/P_{sat} = 0.3 – 0.7).

Since the FTIR analysis was performed directly on the coated silicon wafers, a background scan was done on the bare silicon wafer before performing the measurement. The FTIR spectrum of the monomer was also checked for comparison with the iCVD polymer films. This measurement was done with the same machine, but equipped with a Specac Golden Gate reflector ATR system with a diamond tip and by using the same conditions.

S.3.2. Proton nuclear magnetic resonance (^{1}H NMR)

^{1}H NMR was performed on a 500 MHz Varian Unity INOVA NMR at room temperature. The samples were prepared by dilution in deuterated DMSO-d_6. Single-pulse experiments were performed using 5-sec relaxation delay, 3-sec acquisition time and 128 scans. All spectra were processed by MestReNova 12.0 software. ^{1}H NMR (DMSO-d_6, 500MHz) of PMBL: δ 4.2 (br, 2H,

CH_2), δ 2.00 (br, 4H, CH_2, CH_2). Figure S5 shows ^1H NMR of two PMBL samples which match with literature data.[2]

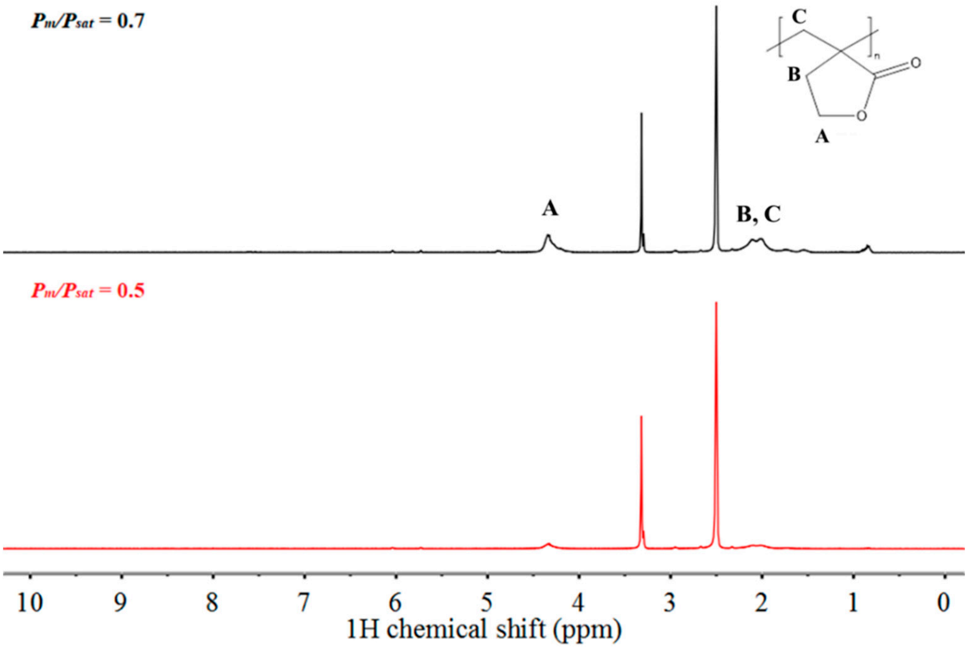

Figure S5. Stack plot for the ^1H NMR spectrum of PMBL samples.

S.3.3. Size-exclusion chromatography (SEC)

SEC was used to measure molecular weights of the polymer using a GPCMax system from Viscotek equipped with 302 TDA detectors array as well as two columns in series (Column Set: Polargel L + M, both 8 µm 30 cm) from Agilent Technologies. The detectors and columns were kept at 50 °C and the solvent containing DMF with 0.01M LiBr was used as an eluent (flow rate - 1 mL/min). To gather material in a powdered form for this characterization technique, the depositions were done directly on the iCVD stage and the solid polymer material was collected from it by scraping with a brass instrument. For polymer sample preparation, the obtained PMBL coating was dissolved in DMF with 0.01M LiBr at a concentration of ≈ 5 mg/mL and ran through a 0.2 µm nylon filter prior to injection. For calibration, near monodisperse PMMA standards from

Polymer Standard Services were used and the resulting data was processed using the OmniSEC software to calculate the number-average molecular weight (M_n), the weight-average molecular weight (M_w), and the polydispersity index (PDI). Figure S6 show the RI trace molecular weight distributions (MWDs) of some samples.

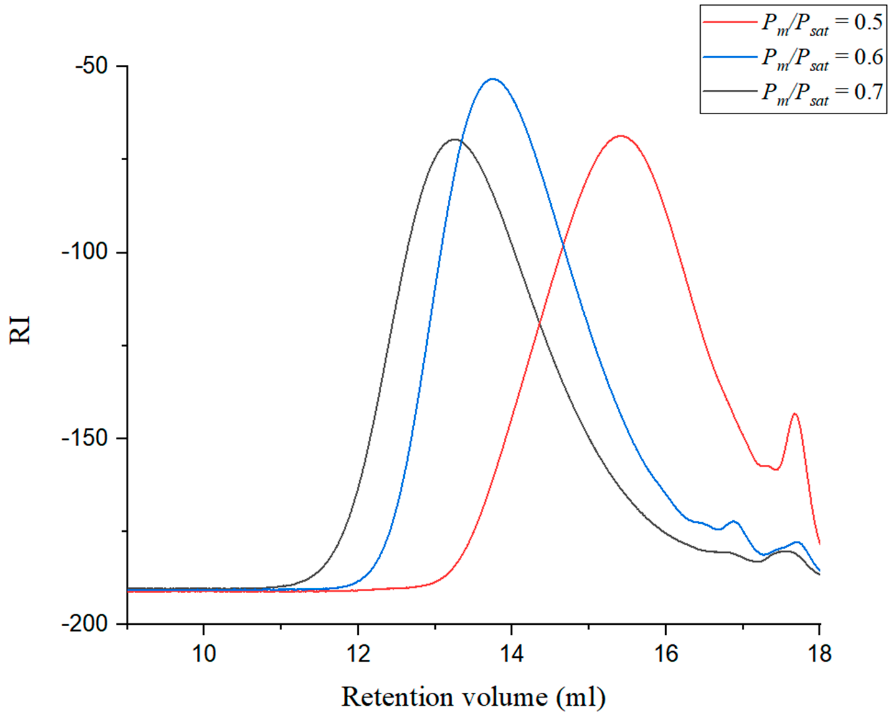

Figure S6. MWDs of PMBL (obtained via SEC using PMMA standards): For the samples P0.52a, P0.61a, P0.70a in Table 1).

S.3.4. Thermogravimetric analysis (TGA)

Samples (1 - 1.5 mg) were prepared in ceramic crucibles. On a Perkin Elmer TGA 4000 instrument, they were heated under a nitrogen atmosphere at a rate of 10 °C min-1 from 35 °C to 900 °C (gas flow rate - 30 ml/min). The resulting data were processed using the Pyris series TGA 4000 software.

S.3.5. Differential scanning calorimetry (DSC)

DSC was performed to study the thermal behaviour of the polymer. The heat flow describes the amount of heat provided per unit time. Samples (≈ 2.5 mg) were prepared in Tzero aluminium pans and placed in a DSC2A-01460 TA instrument of the DSC25 type equipped with a cooler and

an auto sampler. The specimens were exposed to heating and cooling under a 50 $mLmin^{-1}$ flow of nitrogen gas. The procedure started with heating up to 200 °C at a rate of 10 °C min^{-1}, equilibrated at this temperature for 5 minutes, cooled to 25 °C followed by another 5-minute isotherm, and then heated to 200 °C. All T_g values were obtained from the second heating cycle and analysed by taking the midpoint of the slope of the peak via TRIOS software (v5.1.1.46572).

S.3.5.6. UV-vis spectrophotometry (UV-vis)

UV-vis was employed to assess the coating transparency on polycarbonate (PC) and microscopic glass substrates. It was performed on an Agilent CrossLab Cary 60 version 2.0 type spectrophotometer. The samples were measured by attaching the substrates to a specific sample holder directly into the machine chamber and taking the measurement at a 24000 nm/min scanning rate. The spectra were measured in the 200 – 800 nm wavelength region.

S.3.5.7. Nanoindentation

It was carried out using an MTS Nanoindenter XP equipped with a Berkovitch tip. For this characterization technique, a nanoindenter was used to make small indents on the surface of the PMBL coating of different thicknesses on glass as well as silicon wafer substrates, while registering the displacement and reaction forces with high resolution, allowing for the measurement of various properties. The purpose of this technique was to measure the elastic modulus of the polymer coating. The equipment was operated in Continuous Stiffness Measurement mode with a constant nominal strain rate of 0.02 s^{-1}. The depth limit was set as no more than 10% of the film's thickness in order to avoid substrate effects. Twenty-five indentations were made in each sample. Both the elastic modulus and hardness were calculated from the unloading section and averaged. The samples were prepared according to the procedure mentioned above and a summary of the specific samples used, including the coating thicknesses can be found in Table S2 below. These samples were chosen solely based on their thickness, and not according to the P_m/P_{sat} values. Thus, a range of different samples with various thicknesses was chosen to improve the accuracy of the nanoindentation results.

Table S2. Nanoindentation results

		Substrate			SD		SD

Sample name	Film thickness (μm)	type	Depth limit (nm)	**Modulus From Unload (GPa)**		Hardness From Unload (GPa)	
P0.6a’	1.00	glass	100	12.68	0.82	0.71	0.034
P0.7a’	1.70	Si	150	12.63	0.49	0.59	0.014
P0.6b’	6.50	Si	250	9.97	0.30	0.49	0.013
P0.6c’	5.20	glass	250	10.28	0.41	0.51	0.012
P0.6d’	2.6	Si	250	13.17	0.24	0.506	0.009

Nanoindentation was used in order to measure the elastic modulus of the PMBL thin films. Since the depth limit of the nanoindenter was set as no more than 10% of the thickness of the film, the effects of the substrate were assumed as avoided. Following twenty-five indentations for each sample, elastic modulus and hardness were calculated from the unloading section (raw load vs displacement curve included in the Figure S7 and Figure S8, and averaged. The elastic modulus and hardness were calculated for coatings of different thicknesses and on different substrates and are summarized in Table S2. An outstanding result was determined for the elastic modulus measurements and depicted in Figure S9. Hardness results can be observed in Figure S10. In general, no specific trend was observed based on the thickness of the coating and an average modulus from unload was calculated as 11.75 GPa. This value is more than five times higher compared to an earlier reported value of the modulus for PMBL obtained via solution polymerization.[3]

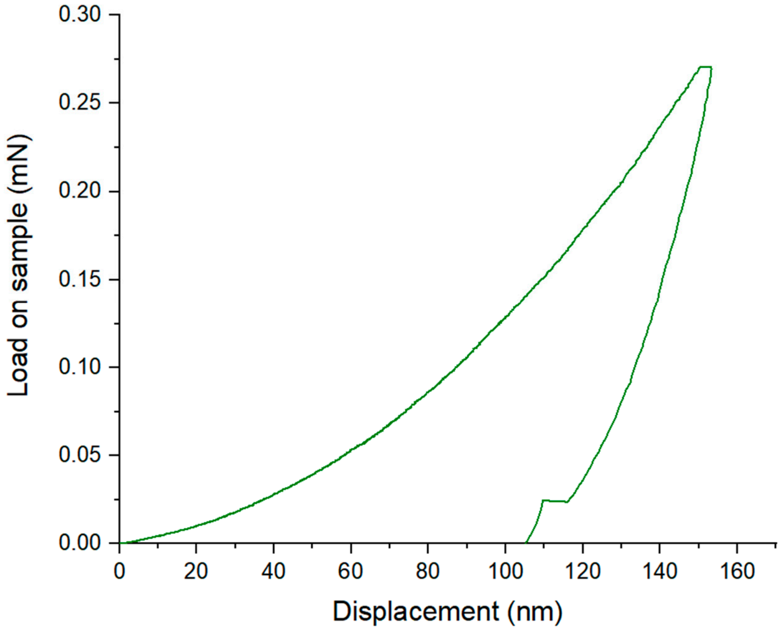

Figure S7. Mechanical properties: nanoindentation data – Displacement vs load curve.

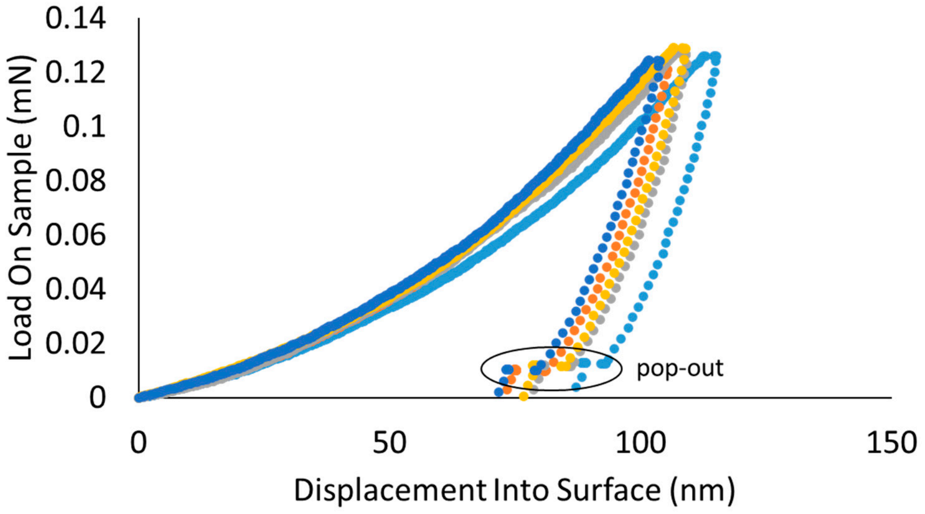

Figure S8. Mechanical properties: Displacement vs load diagram (five randomly chosen curve compilation).

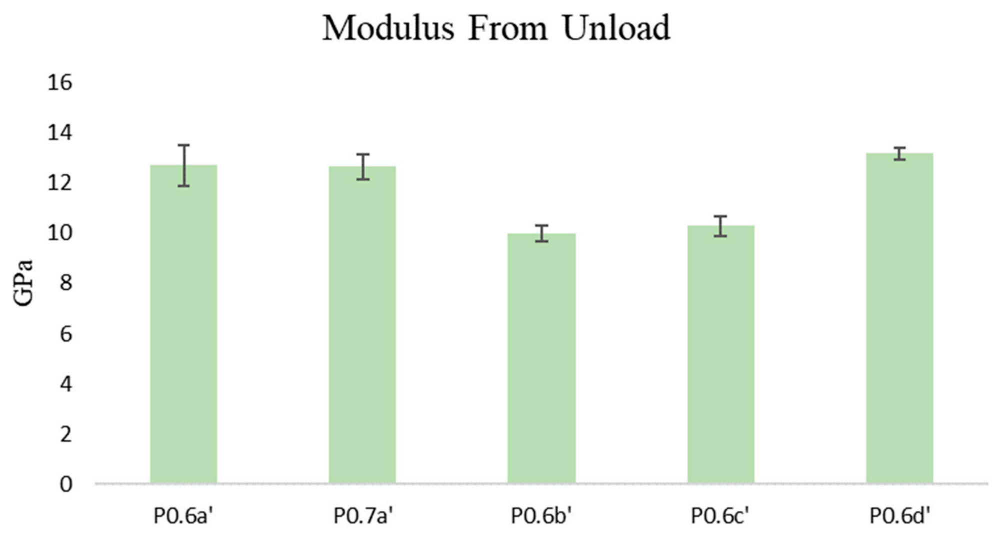

Figure S9. Nanoindentation results for multiple samples: PMBL modulus.

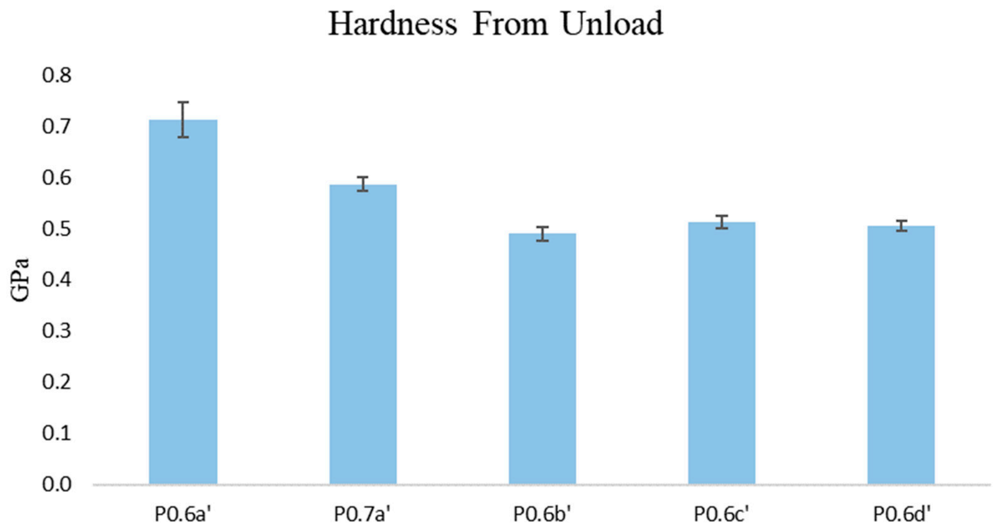

Figure S10. Nanoindentation results for multiple samples: PMBL hardness.

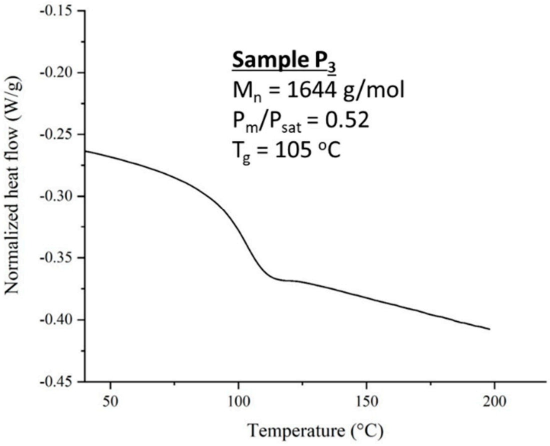

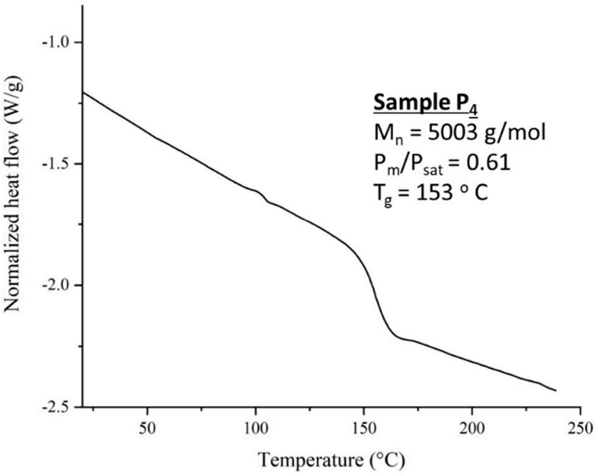

Figure S11. DSC data for samples P3 and P4.

References

(1) Trotta, J. T.; Jin, M.; Stawiasz, K. J.; Michaudel, Q.; Chen, W-L.; Fors, B. P. Synthesis of Methylene Butyrolactone Polymers from Itaconic Acid. *J. Polym. Sci.*, *Part A: Polym. Chem*. **2017**, *55*, 2730–2737. https://doi.org/10.1002/pola.28654.

(2) Mosnáček, J.; Matyjaszewski, K. Atom Transfer Radical Polymerization of Tulipalin A: A Naturally Renewable Monomer. *Macromolecules*. **2008**, *41*, 5509-5511. https://doi.org/10.1021/ma8010813.

(3) Agarwal, S.; Jin, Q.; Maji, S. Biobased Polymers from Plant-Derived Tulipalin A, In Biobased Monomers, Polymers, and Materials, vol. 1105, P. B. Smith and R. A. Gross, Eds. Washington, DC: American Chemical Society, **2012**, 197–212. doi:10.1021/bk-2012-1105.ch013.
